# Integrated Application of Multivariate Statistical Methods to Source Apportionment of Watercourses in the Liao River Basin, Northeast China

**DOI:** 10.3390/ijerph13101035

**Published:** 2016-10-21

**Authors:** Jiabo Chen, Fayun Li, Zhiping Fan, Yanjie Wang

**Affiliations:** 1 National & Local United Engineering Laboratory of Petroleum Chemical Process Operation, Optimization and Energy Conservation Technology, Liaoning Shihua University, Fushun 113001, China; zhiping_fan@hotmail.com (Z.F.); yanjie_wang0810@hotmail.com (Y.W.); 2 Institute of Eco-Environmental Sciences, Liaoning Shihua University, Fushun 113001, China

**Keywords:** spatial and temporal patterns, source apportionment, Liao River, geographic information system (GIS), multivariate analysis

## Abstract

Source apportionment of river water pollution is critical in water resource management and aquatic conservation. Comprehensive application of various GIS-based multivariate statistical methods was performed to analyze datasets (2009–2011) on water quality in the Liao River system (China). Cluster analysis (CA) classified the 12 months of the year into three groups (May–October, February–April and November–January) and the 66 sampling sites into three groups (groups A, B and C) based on similarities in water quality characteristics. Discriminant analysis (DA) determined that temperature, dissolved oxygen (DO), pH, chemical oxygen demand (COD_Mn_)_,_ 5-day biochemical oxygen demand (BOD_5_), NH_4_^+^–N, total phosphorus (TP) and volatile phenols were significant variables affecting temporal variations, with 81.2% correct assignments. Principal component analysis (PCA) and positive matrix factorization (PMF) identified eight potential pollution factors for each part of the data structure, explaining more than 61% of the total variance. Oxygen-consuming organics from cropland and woodland runoff were the main latent pollution factor for group A. For group B, the main pollutants were oxygen-consuming organics, oil, nutrients and fecal matter. For group C, the evaluated pollutants primarily included oxygen-consuming organics, oil and toxic organics.

## 1. Introduction

Surface water quality and aquatic ecosystems have been seriously impacted by complex human activities and natural processes at both river and basin scales, including domestic wastewater, industrial sewage, runoff, land reclamation, oil development, mining exploitation, atmospheric deposition and climate change [1,2,3,4]. Because of the complexity of river water environments [5,6] and obvious differences in regional pollution characteristics [2,4,5], regulators and experts of environmental protection face severe challenges in preventing and controlling water pollution. Accordingly, understanding the spatial and temporal patterns in the hydrochemistry of river water [1,7], extracting the most useful information from complicated monitoring data [2] and identifying the major sources of regional water pollution [3,5] can aid regulators in establishing priority measures for the efficient conservation and restoration of river water resources and aquatic ecosystems. 

The Liao River is situated in northeast China and is one of seven major rivers in China. The Liao River is one of the most seriously polluted rivers in China [8]. In the past two decades, the rapid development of industry and agriculture has profoundly changed environmental conditions in the Liao River basin, particularly in the middle and lower reaches [9]. The rapid development of heavy industry (such as energy, petrochemical, metallurgy, machinery and building material production) in the Liao River basin has made a great historical contribution to the rapid growth of urbanization and industrialization in China [10,11,12]. However, the conflict between the use of water for production and residents caused by rapid economic growth versus use for ecological water has had a negative effect on water quality in Liao River basin (Northeast China), and the level of surface water utilization in the Liao River basin reached 77% in 2000. Furthermore, the urban areas surrounding the river discharge large amounts of water pollutants (including 130.265 × 10^4^ t/a COD (chemical oxygen demand) and 13.267 × 10^4^ t/a NH_4_^+^–N in 2000), causing deterioration of surface water quality [13]. Degradation of the water environment not only hinders sustainable societal development but also endangers human health and aquatic life [3].

Pearson correlations are currently widely employed in evaluating the relationships among river water quality parameters [5]. Clustering analysis (CA) is applied to group objects into categories based on their similarity through an unsupervised multivariate technique [4,6], whereas discriminant analysis (DA) provides statistical classification of samples and helps group samples with common properties [6,14]. Principal component analysis (PCA) and factor analysis (FA) have been used to determine latent sources of pollution, while effectively reducing data dimensionality with minimal loss of meaningful information and grouping multiple variables according to their common characteristics in studies on water environments [4,6,15,16]. The positive matrix factorization (PMF) approach has been successfully used to quantitatively apportion concentrations to their sources [17] and demarcate major sources of pollution [18].

Pearson correlations, CA, DA, PCA, FA and PMF have been effectively applied for assessment of the spatial and temporal variations in surface water and estimation of latent pollution factors [2,3,5,6]. These show the reliability and feasibility of the above multivariate statistical techniques in the research and management of river water environment. 

Several commonly applied statistical techniques are used for pollution source apportionment, with their own advantages and limitations [5]. A summary of relevant statistical methods employed in recent years is provided in several publications [3,5,16]. Some recent publications [2,3,6,19,20] have described the application of different statistical analysis techniques, such as CA, DA, PCA and PMF, to explore the spatial and temporal patterns of water quality and determine latent pollution sources in studies on the water environment in China. However, few of these works have been able to geographically link water pollution with specific anthropogenic activities, which can then be applied to guide strategies for the protection of water resources and aquatic ecosystems. Few studies have used multivariate statistical methods to obtain the pollution characteristics of key areas of the Liao River basin (China) based on the geographic information system (GIS) environment. 

In this study, we expanded on previous research [5] via the integrated application of various GIS-based multivariate statistical methods with large datasets obtained during a 3-year (2009–2011) water quality monitoring program to investigate latent pollution sources for the Liao River basin, Northeast China. The main purpose of this study was to identify the main factors involved in the pollution of the Liao River system. These results could be helpful for more effectively developing river water pollution control strategies for the Liao River system. 

## 2. Materials and Methods 

### 2.1. Study Area and Monitoring Sites

**Figure A1:**
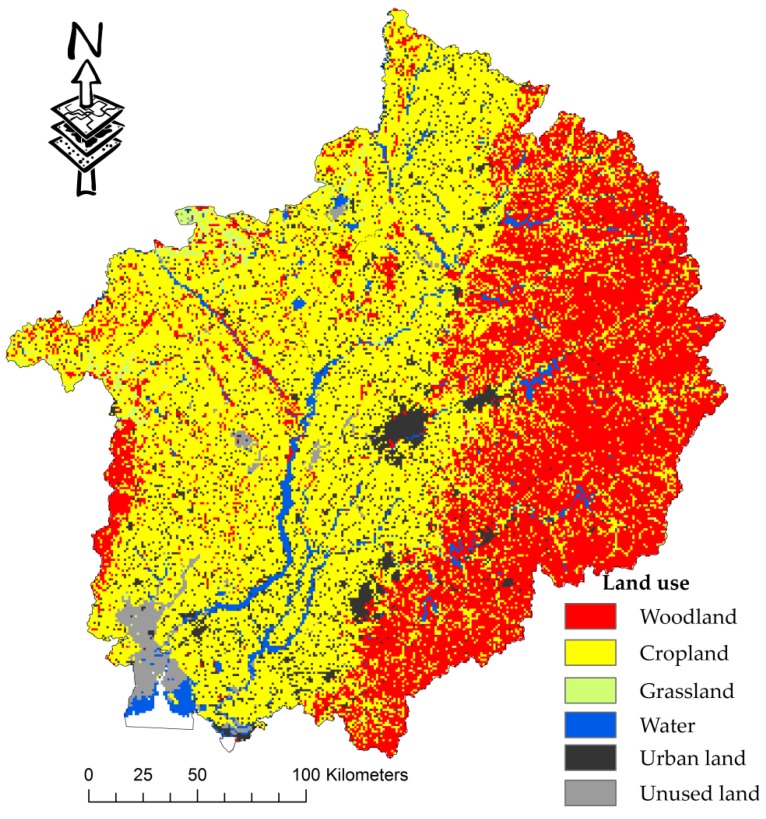
Land use map of the study area. *Note:* the data set was provided by Data Center for Resources and Environmental Sciences, Chinese Academy of Sciences (RESDC).

The Liao River basin, including the majority of Liaoning Province and parts of the Inner Mongolia Autonomous Region and Jilin and Hebei Provinces, covers an area of approximately 21.96 × 10^4^ km^2^, extending from 40°30′ to 45°10′ N and 117°00’ to 125°30’ E [21]. The Liao River basin is located in the temperate and warm temperate belt and is the monsoon climate [9]. The mean annual temperature in this area is 9.4 °C; January is the coldest month during the year, and July is the hottest. Total annual rainfall is approximately 628 cm, and the amount of rainfall during monsoon months (May–September) accounts for more than 80% of the total annual rainfall. The Liao River system comprises two main rivers (the Liao River and Daliao River). The Liao River system exhibits a main stream of more 513 km in length and over 40 tributaries. The water in the middle reaches of the Liao River mainly comes from the East Liao River (the main tributary of the Liao River), where there is sufficient precipitation and a high percentage of vegetation coverage (more than 60% of the East Liao River watershed area, Figure A1) [9,22,23]. The Daliao River has two main tributaries (the Hun River and the Taizi River), and its basin has been affected by the rapid development of heavy industry in Northeast China. The Daliao River basin encompasses several large and mid-sized cities, such as Shenyang city, which is a super city and is the capital of Liaoning Province [24,25]. The upstream areas of the Liao River basin mainly consist of woodland and grassland (more than 80% of the upstream watershed area of the Liao River basin, Figure A1). The middle and downstream areas of the Liao River basin mainly consist of cropland (more than 85% of the middle and downstream watershed area, Figure A1), with scattered urban land (less than 10% of the middle and downstream watershed area, Figure A1) [9,25,26]. In 2005, the population of the Liao River basin was approximately 3500 × 10^4^, and gross domestic product (GDP) production was approximately 6000 billion Yuan. The majority of the population and GDP production is centered in towns and cities in this area. The GDP per capita in the Liao River basin is higher than the national average [9]. The 66 water quality sampling sites examined in this study covered a wide range of key areas of the Liao River system in Northeast China to reasonably represent river water quality.

### 2.2. Data Sources

**Figure 1:**
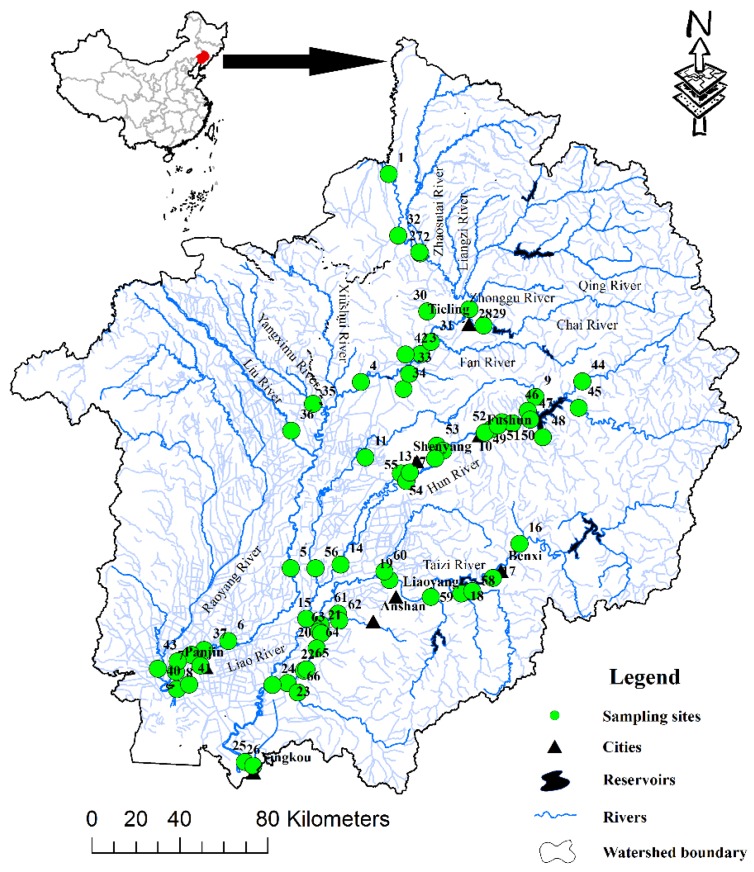
Study area and water quality sampling sites.

Datasets for 66 sampling sites (Figure 1) including 13 typical variables analyzed monthly for three years (2009–2011) were provided by the Environmental Protection Bureau of Liaoning Province. The samples were collected once a month between 9:00 am and 16:00 pm. The chemical analysis was performed in the laboratory within 24 h of collecting the water samples. The monitored parameters included temperature, dissolved oxygen (DO), pH, chemical oxygen demand (COD_Mn_), 5-day biochemical oxygen demand (BOD_5_), ammonical nitrogen (NH_4_^+^–N), total phosphorus (TP), mercury (Hg), lead (Pb), volatile phenols, petroleum, fecal coliforms (*E. coli*) and electrical conductivity (EC). The sampling, preservation and analytical procedures were performed according to national standard methods for China [27]. Analytical methods for water quality parameters are listed in Table A1. Hydrological data (streamflow discharge) for 10 years (2000 and 2002–2010, Liaozhong Gauging Station) were obtained from the Hydrological Bureau of Liaoning Province. The land use data set was provided by Data Center for Resources and Environmental Sciences, Chinese Academy of Sciences (RESDC) [28].

### 2.3. Statistical Analysis

#### 2.3.1. Data Treatment

The following data pretreatment measures were applied: (1) Missing data were evaluated based on average values from the corresponding datasets [16,20]; (2) When water quality parameter values (<1%) were below the minimum detection limits, the values were set to the detection limits [29]; (3) The normality of the distribution of each water quality parameter was checked through analysis of kurtosis and skewness before applying the multivariate statistical analyses [6,16,30]. After log-transformation of the data, the their skewness and kurtosis were significantly reduced; these variables (with the exception of DO, COD_Mn_, NH_4_^+^–N, TP, petrol, Hg, Pb and EC) showed values ranging from −0.723 to 0.44 and from −1.002 to 1.252, respectively; (4) Datasets were standardized (mean = 0, variance = 1) when using CA and PCA to minimize the effects of dimension and differences in the variance of water quality parameters [1,16,30]; and (5) the Kaiser-Meyer-Olkin (KMO) measure and Bartlett’s sphericity tests were used to evaluate the suitability of the datasets prior to PCA [20]. 

PMF analysis of the datasets was performed using the EPA PMF 5.0 program (Environmental Protection Agency, Washington, DC, USA). The other statistical computations were performed with SPSS 19.0 (IBM SPSS, Chicago, IL, USA) for Windows. GIS maps were generated using ArcGIS 10.0 (ESRI, San Diego, CA, USA).

#### 2.3.2. Analysis of Variance (ANOVA)

ANOVA was performed to analyze the significant spatial and temporal differences (*p* < 0.05).

#### 2.3.3. Pearson Correlation 

Pearson correlations are currently widely employed in evaluating the relationships among river water quality parameters [5]. Relationships among the considered water quality parameters were tested using Pearson’s coefficient with statistical significance set at *p* < 0.05.

#### 2.3.4. Cluster Analysis (CA)

CA is a multivariate statistical analysis technique that classifies all dissimilar objects into different groups with an unsupervised pattern based on the characteristics they possess [2,30,31,32]. High internal (within-group) homogeneity and external (between-group) heterogeneity should be observable in the resulting groups of objects [19,33]. CA was used to analyze our dataset to determine the temporal and spatial similarity of river water quality [1,16,20]. We performed hierarchical CA on the standardized dataset using Ward’s method, with squared Euclidean distances as a similarity measure, to present an illustrated dendrogram [1,6,33]. The temporal and spatial variability of water quality in the Liao River basin was evaluated based on hierarchical CA using linkage distance [6,20], and the (D_link_/D_max_) ratio between the linkage distance for a particular case (D_link_) divided by the maximal linkage distance (D_max_) was used to standardize the linkage distance [1,33,34].

#### 2.3.5. Discriminant Analysis (DA)

DA was performed to classify samples exhibiting similar properties with prior knowledge of objects and to identify the most significant discriminant variables for several naturally occurring groups compared with CA [1,33,35]. If DA is effective for a specific data source, the table of classification matrices (including correct and incorrect estimates) will provide a high correct percentage [16,33]. DA was applied in stepwise mode to confirm the groups obtained via CA and to estimate both temporal and spatial variations on the basis of the discriminant variables [16,36]; the sampling periods (temporal variation) and sites (spatial variation) were the clustering (dependent) variables; and all the analyzed water quality parameters were the independent variables [16,20,36].

#### 2.3.6. Principal Component Analysis (PCA)

PCA was used to extract eigenvalues and eigenvectors (loadings or weightings) from the covariance matrix of the original variables to generate new orthogonal (uncorrelated) variables referred to as varifactors (VFs) through VARIMAX rotation; VFs are linear combinations of the original variables [14,19,20,33,37,38,39], and a VF can comprise both potential and hypothetical variables [1,4,39,40]. PCA is usually applied to obtain the minimal number of factors accounting for the maximal variance in the dataset [19,21]. Finally, the few identified factors will usually explain the vast majority of the entire original information [1,33]. PCA was applied to obtain composite variables identified as latent water pollution factors for the Liao River basin in Northeast China.

#### 2.3.7. Positive Matrix Factorization (PMF)

PMF is a multivariate factorization model based on a least squares approach, using a data point weighting method [17,18]. The model can be written as follows in Equation (1):

(1) $$X_{ij}=\sum_{k=1}^{p}g_{ik}f_{kj}+e_{ij}$$

where *X_ij_* represents the elements of the input data matrix of *i* (number of samples) by *j* (chemical species) dimensions; *g_ik_* represents the elements of the factor scores; *f_kj_* represents the factor-loading matrices; *e_ij_* is the residual for each sample/species; and *p* is the number of factors.

The task of PMF is to minimize the objective function, *Q* (Equation (2)), based on the uncertainties [17].

(2) $$Q=\sum_{i=1}^{n}\sum_{j=1}^{m}{\left[\frac{x_{ij}-\sum_{k=1}^{p}g_{ik}f_{kj}}{u_{ij}}\right]}^{2}$$

where *u_ij_* is the uncertainty in the *jth* species for sample number *i*.

## 3. Results

### 3.1. Temporal/Spatial Grouping

**Figure 2:**
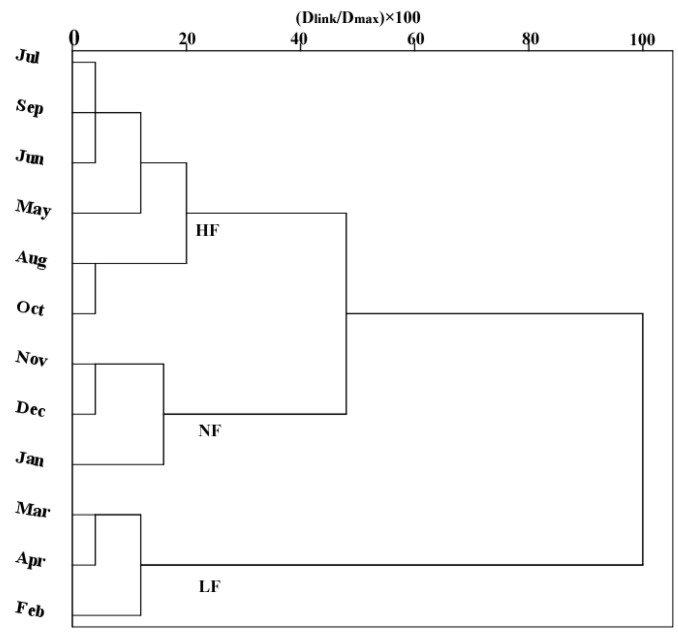
Dendrogram showing the temporal similarities of the monitoring periods produced through cluster analysis. *Note:* HF represents high flow period, NF represents normal flow period, LF represents low flow period.

**Figure 3:**
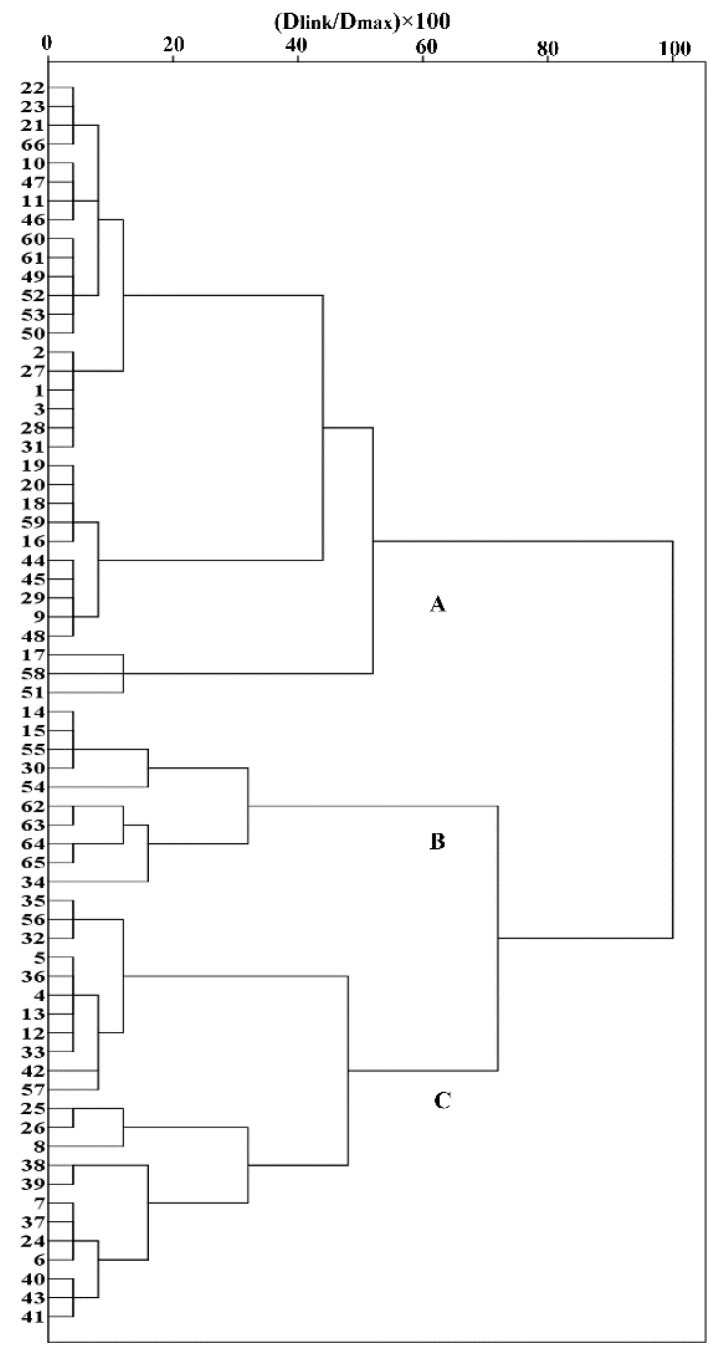
Dendrogram showing the spatial similarities of the sampling sites produced through cluster analysis. *Note:* A, B and C represent different group of sampling sites.

Hierarchical CA was applied to group the water quality dataset based on the temporal and spatial variation (using sampling sites in key areas of the Liao River basin) in river water quality in the resulting dendrogram. There is a seasonal flow change law applying to most rivers in the world, so the flow period division is in accordance with the seasonal flow change of the river [41]. Temporal CA generated a dendrogram (Figure 2) that clearly separated the 12 months of the year into three groups at (D_link_/D_max_) × 100 < 50, with significant differences between the three groups. Group 1 included May–October, which approximately corresponded to the high flow period (HF period) in the Liao River basin. More than 80% of the annual total precipitation falls in this period according to ten years of hydrology data. Group 2 consisted of February–April, which closely corresponded to the low flow period (LF period). Finally, Group 3 comprised November–January, which approximately corresponded to the normal flow period (NF period). A statistical description of discharge that coincides with each type of flow period is listed in Table A2. The spatial CA rendered a dendrogram that grouped all 66 monitoring sites into three different groups at (D_link_/D_max_) × 100 < 80 (Figure 3), similar to the temporal cluster analysis. Group A contained sites S1–S3, S9–S11, S16–S23, S27–S29, S31, S44–S53, S58–S61, and S66; group B comprised sites S4–S8, S12–S13, S24–S26, S32–S33, and S35–S43; and group C contained sites S54–S55, S62–S65, S14–S15, S30 and S34. The spatial CA generated three groups of sampling sites with similar water pollution characteristics in a very convincing manner. In group A, seven sites (S1–S3, S27–S29, and S31) were situated in the upper and middle reaches of the Liao River; 13 sites (S9–S11 and S44–S53) were situated in the upper reaches of the Hun River; and 13 sites were situated in the Taizi River (S16–S23, S58–S61 and S66). In group B, two sites (S30 and S34) were situated in the middle reaches of the Liao River; four sites (S14–S15 and S54–S55) were situated in the lower reaches of the Hun River; and four sites (S62–S65) were situated in the lower reaches of the Taizi River. In group C, sixteen sites (S4–S8, S32–S33 and S35–S43) were situated in the middle and lower reaches of the Liao River; four sites (S12–S13 and S56–S57) were situated in the lower reaches of the Hun River; and three sites (S24–S26) were situated in the lower reaches of the Daliao River.

### 3.2. Temporal/Spatial Variations in River Water Quality

Temporal variations in river water quality were estimated through DA after separating all data for key areas of Liao River basin into three seasonal groups. Temporal DA produced classification matrices (CMs) with 81.2% correct assignments using only eight discriminant parameters (Table 1). Thus, the temporal DA results showed that temperature, DO, pH, COD_Mn__,_ BOD_5_, NH_4_^+^–N, total phosphorus (TP) and volatile phenols were the most significant variables for discriminating between the three periods and that these eight parameters explained most of the temporal variations in the water quality of the Liao River system.

**Figure 4:**
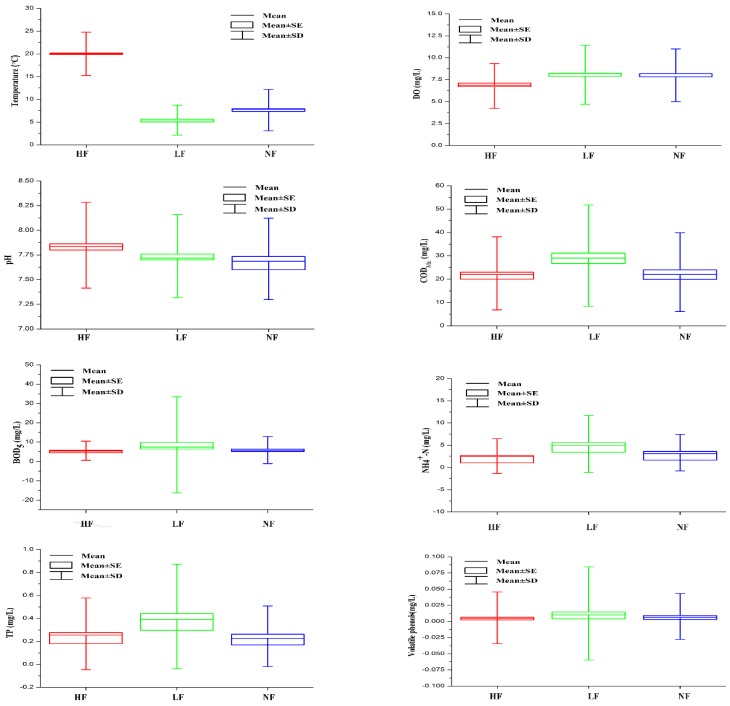
Box and whisker plots of discriminant parameters produced through temporal discriminant analysis. *Note:* SD and SE are the abbreviation of standard deviation and standard error, respectively. DO = dissolved oxygen, COD_Mn_ = chemical oxygen demand, BOD_5_ = 5-day biochemical oxygen demand, TP = total phosphorus.

Box and whisker plots of the selected water quality variables supporting the temporal variations identified through temporal DA are given in Figure 4. The results showed that temperature and pH were generally higher in the HF season than the other seasons, whereas higher values for COD_Mn_, BOD_5_, NH_4_^+^–N, TP and volatile phenols were observed in the LF season than in the other seasons. 

Spatial variations in water quality were evaluated through DA after classifying the data for the study area into three spatial groups. DA also produced CMs with approximately 81.2% correct assignments for the three groups identified by spatial CA (Table 1). The stepwise DA showed that all river water quality parameters were discriminant variables of spatial variation.

**Figure 5:**
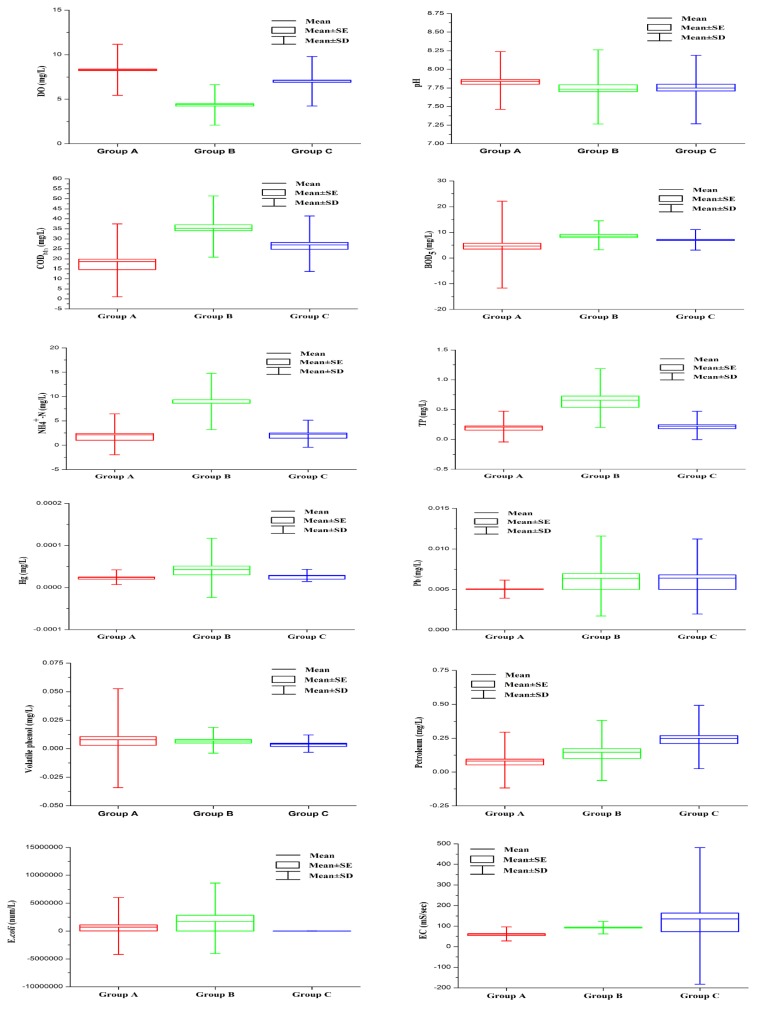
Box and whisker plots of discriminant parameters (not including temperature) produced through spatial discriminant analysis.

**Figure 6:**
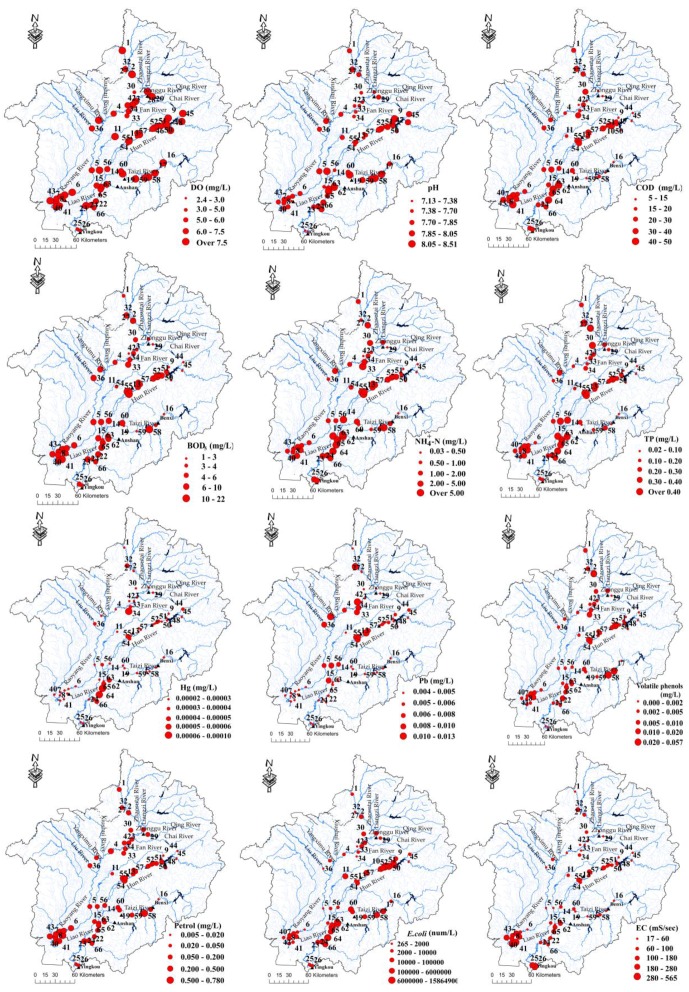
Spatial variations in DO, pH, COD_Mn_, BOD_5_, NH_4_^+^–N, TP, Hg, Pb, volatile phenols, petrol, *E. coli* and EC in the study area. *Note:* EC = electrical conductivity.

Box and whisker plots of the discriminant parameters supporting the spatial variations determined through spatial DA are included in Figure 5. Figure 5 and Figure 6 show that most of the parameters (apart from T, pH, volatile phenols, petrol and EC) presented higher values (DO exhibited an inverse pattern) in group B than in the other two groups. Petroleum and EC were higher in group C than in the other groups, and volatile phenols were higher in group A than in the other groups. COD_Mn_, BOD_5_ and NH_4_^+^–N were higher in urban areas than in nearby rural areas, while DO displayed an inverse trend with the urbanization level [5,37]. Hg was higher in the lower reaches of the Taizi River, whereas Pb was higher in the middle reaches of the Hun River and Liao River. TP was higher in the lower reaches of the Liao River, and *E. coli* was higher in the middle and lower sections of the Hun and Taizi Rivers. The effects of volatile phenols in the Fushun section of the Hun River and the Benxi section of the Taizi River (sites 17, 51 and 58) were greatest in the Liao River system. The EC values were higher in the Liao River Estuary than the other areas (Figure 6).

### 3.3. Identification of Latent Pollution Factors

The 66 monitoring sites were applied to evaluate the correlation matrix of the 13 measured parameters (Table 2). COD_Mn_ was highly correlated with BOD_5_, TP and *E. coli* in groups A and C (*r* = 0.566–0.902, *p* < 0.01). High positive correlations were observed between *E. coli* and NH_4_^+^–N in the all three groups (*r* = 0.374–0.664, *p* < 0.1). 

**Figure 7:**
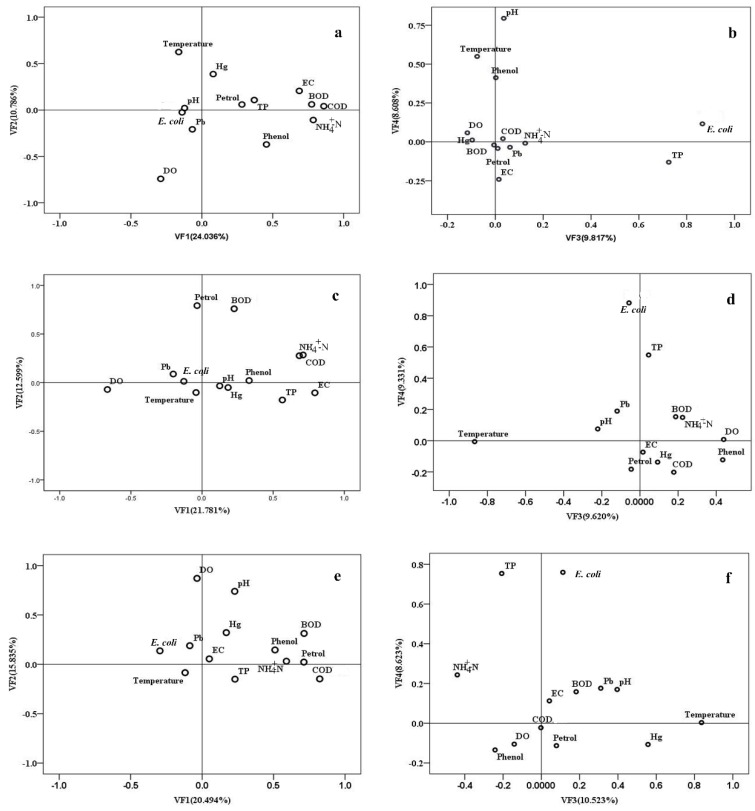
Scatter plot of loadings for the four VFs for group A (**a**,**b**); group B (**c**,**d**) and group C (**e**,**f**).

PCA was used to evaluate the latent pollution factors based on the standardized datasets separately for three different groups (groups A, B and C), as determined via CA (Table 3 and Figure 7). The KMO values for the three groups (groups A, B and C) were 0.709, 0.610 and 0.647, respectively, and the significance levels determined by Bartlett’s sphericity test were all less than 0.001, which showed that the PCA was useful for significantly reducing the dimensionality of the data [1,16,20,33]. The PCA with VARIMAX rotation produced 5–6 VFs (eigenvalues equal or greater than 1) and explained 61.217%, 69.645% and 63.57% of the total variance in groups A, B and C, respectively. PCA results (including the loading of the 13 water quality parameters, the variance contribution rate of each VF and the accumulated variance contribution rate) for the three groups are listed in Table 3. Some studies [6,33] classify factor loading values of 0.50–0.30, 0.75–0.50 and >0.75 as “weak”, “moderate” and “strong”, respectively, corresponding to the absolute loading. The VF loading plot (Figure 7) of the three different groups (groups A, B and C) revealed relationships among the river water quality variables, with a shorter distance corresponding to a stronger correlation between the parameters [6,16,20,29,33].

For group A (Table 3 and Figure 7), VF1, which explained 24.036% of the total variance, exhibited strong positive loading on only COD_Mn_, BOD_5_ and NH_4_^+^–N and moderately positive loading on EC. Thus, VF1 represented oxygen-consuming organic pollution from non-point pollution caused by nutrient runoff from cropland and woodland [6,25]. VF2 (accounting for 10.786% of the total variance) displayed moderately positive loading on temperature and moderately negative loading on DO and was attributed to seasonal changes [14,16,20]. Additionally, VF3 (explaining 9.817% of the total variance) presented strong positive loading on *E. coli* and moderately positive loading on TP and may be interpreted as fecal and nutrient (TP) pollution originating from local livestock farms and domestic wastewater [10]. VF4, accounting for 8.608% of the total variance, exhibited strong positive loading on pH and moderate positive loading on temperature and may be interpreted as the physicochemical source of the variability [16,20,33,34]. VF5 (explaining 7.971% of the total variance) showed strong positive loading on Pb and moderately positive loading on Hg and was subject to heavy metal pollution originating from mining activity [10]. For group B (Table 3 and Figure 7), VF1 (explaining 21.781% of the total variance) presented strong positive loading on EC, moderately positive loading on NH_4_^+^–N and TP, and moderately negative loading on DO, likely representing nutrient pollution from domestic wastewater and sewage treatment works [23,26,42]. VF2 (accounting for 12.599% of the total variance) exhibited strong positive loading on Petrol and BOD_5_ and, thus, represented oil pollution originating from the petroleum chemical industry [2,12,42,43]. Additionally, VF3 (explaining 9.62% of the total variance) showed strong negative loading on temperature, similar to VF2 of group A, representing natural source impacted by seasonal changes. VF4 (accounting for 9.331% of the total variance) presented strong positive loading on *E. coli* and moderately positive loading on TP, similar to VF3 of group A. VF5 (explaining 8.28% of the total variance) exhibited strong positive loading on Pb and moderately positive loading on Hg and was attributed to heavy metal pollution from industrial sewage [42,44]. For group C (Table 3 and Figure 7), VF1 (accounting for 20.494% of the total variance) showed strong positive loading on COD_Mn_ and moderately positive loading on BOD_5_, NH_4_^+^–N, Petrol and Volatile phenols, which represented mixed pollution, including oil.

Pollution, oxygen-consuming organic pollution and toxic organic pollution. Oil pollutants originated from oil production and the petroleum chemical industry, whereas oxygen-consuming/toxic organics mainly originated from steel-making, gas-firing, cooking water, industry, domestic wastewater, garbage produced by humans and bilge water [10,12,42,44]. VF2 (explaining 15.835% of the total variance) presented strong positive loading on DO and moderately positive loading on pH, similar to VF4 of group A (physicochemical sources). VF3 (accounting for 10.523% of the total variance) exhibited strong positive loading on temperature and moderately positive loading on Hg and was attributed to heavy mental pollution originating from industrial sewage during different flow periods [1,2,6,44]. VF4 (explaining 8.623% of the total variance) showed strong positive loading on *E. coli* and TP, similar to VF3 of group A. 

**Figure 8:**
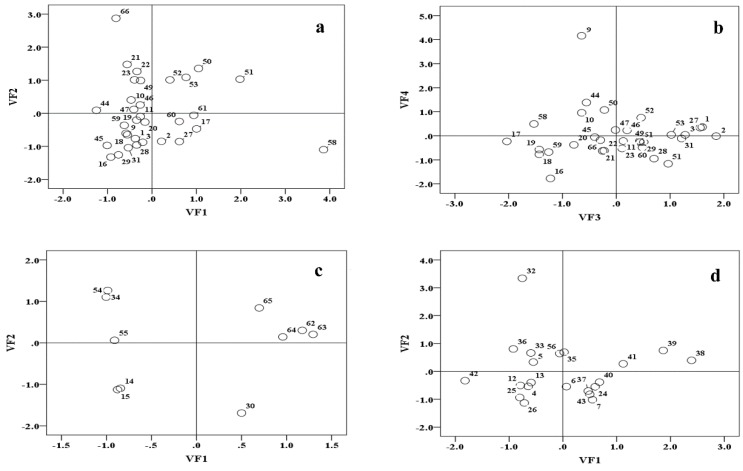
Scatter plot of the scores for the VFs for group A (**a**,**b**); group B (**c**) and group C (**d**). *Note*: Each number was short for each sampling site.

To identify the spatial patterns in latent pollution factors, the loadings and scores of the VFs were plotted [1,6,16,45] for three different group (groups A, B and C) of monitoring sites to illustrate spatial differences (Figure 8). The larger VF scores presented a greater effect [2,6,16,20,33]. In group A (Figure 8a,b), some sites (e.g., 58, 50, 51, 17 and 61) were strongly influenced by organic pollution, whereas other sites (e.g., 2, 1, 27, 3, 31, 53 and 28) were primarily influenced by nutrient pollution. In group B (Figure 8c), some sites (e.g., 62, 63 and 64) were predominantly influenced by nutrient pollution. In group C (Figure 8d), some sites (e.g., 38, 39, 40 and 41) were strongly influenced by oil pollution. 

PCA was also applied to the datasets from three different periods (HF, LF and NF) for each group (A, B and C) of sampling sites to consider the influence of temporal variation on the VFs. The results (Table 4) for KMO and Bartlett’s test showed that PCA was effective in reducing dimensionality for all datasets from the Liao River system. The statistical analysis procedures were the same as the previous PCA. Table 5 summarizes the results of source identification for the monitoring sites (groups A, B and C) in the different periods. Most sampling sites in group A were not obviously affected by heavy metal pollution during the NF period. In group B, most sampling sites were influenced by toxic organic pollution during HF and NF periods.

## 4. Discussion

### 4.1. Temporal/Spatial Similarities and Groupings

The temporal variation in water quality (Figure 2) in the Liao River system was clearly affected by hydrologic conditions (high, normal and low flow periods) and also by seasonal changes and river water pollution characteristics to some degree [2,6,16,20,33]. As shown by the results (Figure 3) of spatial CA, the sites in group A were primarily situated in the upper and middle reaches of the Liao, Hun and Taizi Rivers. The most upstream sites in the study area were located in a timbered mountainous region receiving little influence from human activities [9,25]. In group B, the sites were situated in the middle reaches of the Liao River and the lower reaches of the Hun and Taizi Rivers, which pass through the areas showing the highest population density and greatest industrialization within the Liao River watershed and are subject to serious river water pollution problems [9,10,11,12,26,43,44]. The sites in group B primarily received discharge from industrial sewage, domestic wastewater and sewage treatment works in city areas and non-point source pollution in rural areas [10,11,14,21,25,26]. The sites in group C were situated in the middle and lower reaches of the Liao River and the lower reaches of the Hun and Daliao Rivers, which are primarily influenced by oil production and petrochemical industry pollution [12,43,44,46,47]. The results of temporal and spatial CA showed that the monitoring frequency and number of monitoring sites may be appropriately reduced through selecting monitoring periods in different seasons and sampling sites from different groups [1,2,20]. 

### 4.2. Temporal/Spatial Variations in River Water Quality

The characterization of seasonal and spatial variations in water quality is important for evaluating river pollution caused by anthropogenic or natural factors [5,6,16,37]. Temperature and pH were generally higher, while DO was generally lower in the HF season (20.0 °C for water temperature, 7.85 for pH and 6.78 mg/L for DO) than the other seasons (6.5 °C for water temperature, 7.72 for pH and 8.03 mg/L for DO), whereas higher COD_Mn_, BOD_5_, NH_4_^+^–N, TP and volatile phenols values were observed in the LF season (30.07 mg/L for COD_Mn_, 8.61 mg/L for BOD_5_, 5.289 mg/L for NH_4_^+^–N, 0.417 mg/L for TP and 0.0123 mg/L for Volatile phenols) than in the other seasons (22.73 mg/L for COD_Mn_, 5.75 mg/L for BOD_5_, 2.451 mg/L for NH_4_^+^–N, 0.2556 mg/L for TP and 0.0066 or mg/L for Volatile phenols) (Figure 4 and Table A3). The lower DO values recorded during the HF period were due to many factors. For example, the local climate differed between seasons, with an obviously higher mean temperature occurring during the HF period (May–October) than the other periods (Figure 4); thus, the lower DO in the river water observed in summer than in the other seasons results from a natural process because warm water shows lower saturation values for dissolved oxygen and is able to hold less dissolved oxygen [1,5,16,20,48]. Additionally, intense rainfall washes continental organic matter (such as agricultural, forestal and municipal wastes) into the surface water, and organic matter consumes a large amount of dissolved oxygen through biodegradation [6,21,25]. Lower NH_4_^+^–N, volatile phenol and petrol concentrations were detected during the HF period, due to the effect of dilution by rainfall on point source pollution [2,3,6,42,43,46,47]. The average COD_Mn_, BOD_5_ and TP concentrations were all higher during the LF period than the NF and HF periods due to a lower flow (which would dilute oxygen-consuming organics and nutrients). Hg, Pb and *E. coli* displayed no statistically significant differences (Figure 4) among the three periods, which was attributed to the relatively low values of these variables and the investigation of sampling sites with similar sources during the different periods [21,42]. 

Among the measured water quality variables, most of the variables in group A (sites 9, 16, 44, 45 and 48) exhibited low values because these areas were nearly pristine, without significant point source pollution (Figure 6) [10,21,25]. Within the Liao River basin (Figure 6), the average concentrations of volatile phenols were highest in the Fushun section of the Hun River and the Benxi section of the Taizi River (sites 17, 51 and 58), coming from industrial effluents of the organic chemical industry and steel-making and coke plants [2,10,42,46,47]. Higher COD_Mn_, BOD_5_, NH_4_^+^–N, TP, and Hg values and lower DO and pH values were found in group B (Figure 5 and Figure 6 and Table A3). The higher NH_4_^+^–N, COD_Mn_, and BOD_5_ values and lower pH values were attributed to the fact that most of these monitoring sites were located in watercourses downstream of or near large urban areas in the Liao River basin, where factories are scattered along the low and middle reaches of the Liao and Daliao Rivers [10,12,42,43,46]. Large amounts of incompletely treated domestic and industrial wastewater (domestic wastewater is over 5 × 10^4^ t/a and industrial wastewater is over 4 × 10^5^ t/a) from urban areas are discharged into the Liao River system [10], exceeding the self-purification ability of the river and deteriorating water quality [2,11]. The hydrolysis of some acidic materials from point sources (industrial wastewater) causes a decrease in water pH values [4,6,46]. The higher TP and *E. coli* values observed in most group B sites were primarily due to the fact that the region is a rapidly developing area with the highest population density in the Liao River basin and is characterized by large-scale livestock and poultry breeding production and large areas of cropland [10]. The highest average Hg concentration in the Liao River basin was found in the lower reaches of the Taizi River (Figure 6), due to effluents from industrial wastewater from the cities of Benxi and Anshan [2,10,21,42,44,49,50]. The average Pb concentration in the Liao River basin was higher in the middle reaches of the Hun River and Liao River (Figure 6) because of the surrounding industrial wastewater discharge and mining activities [10,21,25,42,49]. The average *E. coli* concentration was highest in the middle and lower sections of the Hun and Taizi Rivers (Figure 6), which show the highest population densities in the Liao River basin and are characterized by large-scale livestock and poultry breeding farms [10,11,26]. The average petroleum concentration in group C was higher due to oil and gas production and the petrochemical industry. The average EC concentrations at most group C sites were higher in the Liao River Estuary, where the river reaches are affected by tides more often than the other areas [5].

### 4.3. Identification of Latent Pollution Factors

The 66 sampling sites were combined to calculate the Pearson correlation matrix of the 13 analyzed variables (Table 2). The Pearson correlation coefficients should be interpreted with caution due to the simultaneous effects of temporal and spatial variations on river water quality [1,48,51]. However, some hydro-chemical relationships could be inferred [1,26,48]. COD_Mn_ was highly correlated with BOD_5_, TP and *E. coli* in groups A and C, which were responsible for point source pollution. NH_4_^+^–N was highly positively related to *E. coli* in the all three groups, indicating that these pollutants came from similar sources [1,11,16,20,48]. 

EPA PMF software was further used to identify the source of the watercourses in the Liao River basin of Northeast China. The number of source factors should be calculated before running the PMF model [17,18]. Considering the Q Value, the number of source factors for PMF was set to five for three groups (groups A, B and C). 

**Figure 9:**
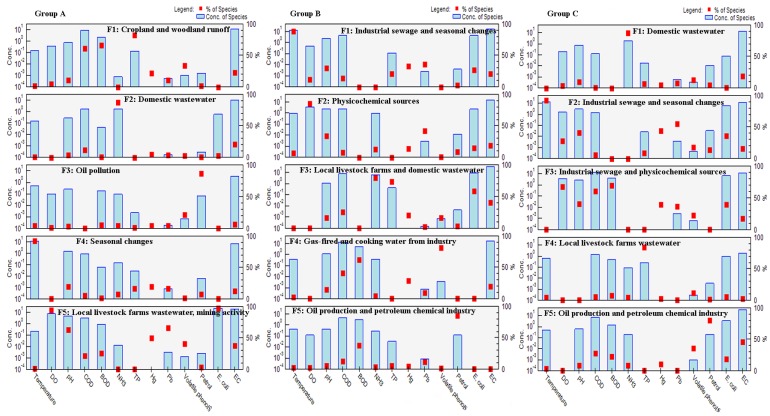
Comparison of factor profiles between the concentration of species and the percentage of species by PMF (Positive Matrix Factorization) for group **A**, group **B** and group **C**.

The concentrations of species and the percentages of each species for the three groups are shown in Figure 9. For group A, factor profile 1 (F1) is dominated by TP, COD and BOD, and it seems reasonable to conclude that F1 represents nutrient and oxygen-consuming organic pollution originating from cropland and woodland runoff [6,25]. Factor profile 2 (F2) was characterized by enrichment with NH_4_^+^–N, and F2 appears to be associated with domestic waste [10,21]. Factor profile 3 (F3) was characterized by enrichment in Petrol, showing an association with oil production at some sampling sites. Factor profile 4 (F4) was dominated by Temperature, and F4 represents seasonal changes [14,16,20]. Factor profile 5 (F5) is dominated by *E. coli*, DO, pH, Pb, Hg and volatile phenols, which appear to be associated with wastewater from local livestock farms, mining activity and the industrial wastewater [10,11,13]. For group B, T, Pb, Hg, pH, TP and *E. coli* dominated in F1, F1 was best suited for industrial sewage and seasonal changes [14,16,20]. F2 was dominated by DO, pH and Pb, which appear to represent physicochemical pollution [16,20,33,34]. F3 was enriched with NH_4_^+^–N, TP and *E. coli*, and it is reasonable to conclude that F3 is associated with local livestock farms and domestic wastewater [10,25,26,42]. Volatile phenols, COD_Mn_ and BOD_5_ dominated in F4, which appear to represent gas-fired and cooking water from industry [2,12,13]. F5 was enriched with Petrol and BOD_5_ and appears to be associated with oil production and petroleum chemical industry [2,12]. For group C, F1 was dominated by NH_4_^+^–N, which appears to be associated with domestic waste [25,26,42]. T, Hg and Pb dominated in F2, which is also associated with industrial sewage and seasonal changes [12,16,20]. F3 was dominated by COD_Mn_, BOD_5_, DO, pH, and which appear to represent oxygen-consuming organic pollution from the industrial sewage and physicochemical sources [25,37,42]. F4 was enriched with TP and might be associated with nutrient pollution from wastewater from local livestock farms. F5 was dominated by Petrol, and it therefore seemed reasonable to conclude that F5 is associated with oil production and petroleum chemical industry [2,12].

The source apportionment results of the PCA and PMF methods are listed in Table 6. Most of the PMF results exhibited good agreement with the PCA results qualitatively, except for F3 (oil pollution) of group A and F4 (gas-fired and cooking water from industry) of group B; however, F3 (oil pollution) of group A and F4 (gas-fired and cooking water from industry) of group B represent continual extensions and refinements of the unexplained variance in their own groups. The results from PMF analysis are in close agreement with the results from PCA method. Compared with PCA, PMF could further quantitatively analyze different pollution sources [17]. However, the results obtained via the PMF method might introduce uncertainty into the conclusions [18,52]. The assessment of source apportionment by the PMF model must be confirmed via PCA to improve its reliability; to a certain extent, the PCA model is the foundation of the PMF, and the PMF model provides more details and expands upon the PCA; the combination of the two methods can provide more valuable information [17,18,52,53]. 

The actual levels and types of river pollution may be determined by many water quality parameters, and each river presents unique characteristics due to the different influence of natural and human activities [3,5,6,16,20,37]. The above 13 variables were selected, and the following eight pollution types were identified in key areas of the Liao River basin: oxygen-consuming organic pollution [2,29] (mainly influenced by non-point sources for group A and point sources for groups B and C, non-point source pollution including agricultural and forestal plant litter and point sources including industrial sewage, domestic wastewater and wastewater treatment plants); toxic organic pollution [2,12,25,33,46,47] (mainly from steel-making, gas-fired and coking plants); nutrients [5,33,54,55] (mainly from non-point sources); fecal pollution [6,12,26] (mainly from livestock and poultry breeding and domestic sewage); heavy metals [2,6,21,49,56,57] (mainly from mining development and industrial sewage); oil pollution [2] (mainly from oil development); physicochemical pollution [16,20,33] (physicochemical sources of the variability); and natural pollution [16,33] (natural sources impacted by seasonal changes). The pollution types at the sampling sites of the three different groups (groups A, B and C) differed markedly during the HF, NF and LF periods (Table 5). The majority of monitoring sites in group A clearly received more heavy metal pollution during HF period than NF and LF periods. Because the areas surrounding some sites in group A were characterized by mineral exploitation activity [10,21,25,50], heavy rainfall carried heavy metals to the surrounding river (e.g., upstream regions of the Hun and Taizi Rivers). Most sites in groups A and C received more nutrient and fecal pollution during the HF period than in the NF and LF periods. The majority of sites in group B showed the inverse pattern, receiving more fecal pollution during the LF and NF periods than the HF period. The sites in group B showed the highest population density and contained many intensive livestock and poultry breeding farms, where a high flow in the HF period diluted fecal pollution; whereas the sites in groups C and A presented lower population densities and less intensive livestock and poultry breeding than group B, and the abundant rainfall in the HF period carried more fecal pollution from non-point pollution sources [10,11,26,42]. Some sites in group C were subject to more serious heavy metal and toxic organic pollution during the NF period than during the HF and LF periods, suggesting that there were more sources of toxic organics and heavy metals during the NF period [9,11,21,25,41,42]. These results of source apportionment considering different periods may be helpful for the prevention and control of water pollution caused by human activities in different seasons [2,16,20,35].

## 5. Conclusions

The comprehensive application of various GIS-based multivariate statistical methods (Pearson correlation, CA, DA, PCA and PMF) was successful in elucidating the spatial and temporal variations of water quality and the source apportionment of water environment pollution in the Liao River system of Liaoning province. The main conclusions were as follows.

(1) In the Liao River basin of Liaoning province, the 12 months of the year could be grouped into three periods (May–October, February–April and November–January), and all sites in the area could be divided into three significantly different groups. It was quite obvious that the CA method was effective in providing a reliable classification of river water in the Liao River basin of Northeast China, and the establishment of an optimal sampling strategy with a lower cost will become possible in the future [2,20].
(2) Temperature, DO, pH, COD_Mn_, BOD_5_, NH_4_^+^–N, TP and volatile phenols were discriminant variables showing temporal variations, with 81.2% correct assignments, and all water quality monitoring parameters were discriminant variables showing spatial variations, also with 81.2% correct assignments.
(3) The patterns of pollution varied significantly on spatial and temporal scales. The results from PMF analysis are in close agreement with the results from the PCA method. For group A, oxygen-consuming organics from cropland and woodland runoff were the main latent pollution source. The main pollutants were oxygen-consuming organics, oil, nutrients and fecal matter for group B. The evaluated pollutants primarily included oxygen-consuming organics, oil and toxic organics for group C.
(4) For group B, the main latent pollution factors were oxygen-consuming organics, oil, nutrients and fecal pollution during the HF and LF periods and oxygen-consuming organics, nutrients, fecal pollution and heavy metals during the NF period. For group C, the main pollutants evaluated mainly consisted of oxygen-consuming organics, oil, and heavy metal during the HF and LF periods and oxygen-consuming organics, toxic organics, oil and heavy metals during the NF period.

## Figures and Tables

**Table 1:** Classification matrices for stepwise discriminant analysis of temporal and spatial variations.

Number of Clusters		Temporal Variation				Spatial Variation			
		%Correct	1st (HF)	2nd (LF)	3rd (NF)	%Correct	1st (A)	2nd (B)	3rd (C)
Three Cluster	1st	57.6	98	58	14	83.4	493	24	74
	2nd	64.9	78	146	1	65.3	13	79	29
	3rd	91.3	50	17	700	83.2	31	19	248
	Total	81.2	226	221	715	81.2	537	122	351

*Note:* HF represents high flow period, NF represents normal flow period, LF represents low flow period.

**Table 2:** Pearson correlation matrix of the 13 analyzed physical-chemical water quality variables.

Parameters		Temperature	DO	pH	COD_Mn_	BOD_5_	NH_4_^+^–N	TP	Hg	Pb	Volatile Phenols	Petrol	*E. coli*	EC
Group A	Temperature	1	-	-	-	-	-	-	-	-	-	-	-	-
	DO	−0.057	1	-	-	-	-	-	-	-	-	-	-	-
	pH	−0.308	0.132	1	-	-	-	-	-	-	-	-	-	-
	COD_Mn_	0.256	−0.739 **	−0.333	1	-	-	-	-	-	-	-	-	-
	BOD_5_	0.009	−0.365 *	−0.068	0.644 **	1	-	-	-	-	-	-	-	-
	NH_4_^+^–N	0.087	−0.490 **	−0.038	0.679 **	0.836 **	1	-	-	-	-	-	-	-
	TP	0.310	−0.703 **	−0.431 *	0.846 **	0.617 **	0.650 **	1			-	-	-	-
	Hg	0.212	−0.634 **	0.033	0.357 *	0.126	0.149	0.388 *	1		-	-	-	-
	Pb	0.073	0.379 *	0.244	−0.157	−0.109	−0.238	−0.321	−0.161	1	-	-	-	-
	Volatile phenols	−0.093	−0.170	0.072	0.385 *	0.637 **	0.475 **	0.394 *	−0.014	−0.121	1	-	-	-
	Petrol	−0.099	−0.187	0.101	0.409 *	0.904 **	0.792 **	0.340	−0.034	−0.116	0.500 **	1	-	-
	*E. coli*	−0.049	−0.556 **	-0.172	0.755 **	0.633 **	0.374*	0.592 **	0.357 *	0.018	0.500 **	0.400 *	1	-
	EC	0.192	−0.616 **	-0.173	0.665 **	0.451**	0.626 **	0.540 **	0.278	−0.143	0.096	0.402 *	0.285	1
Group B	Temperature	1	-	-	-	-	-	-	-	-	-	-	-	-
	DO	0.360	1	-	-	-	-	-	-	-	-	-	-	-
	pH	0.544	−0.104	1	-	-	-	-	-	-	-	-	-	-
	COD_Mn_	−0.102	−0.863 **	0.456	1	-	-	-	-	-	-	-	-	-
	BOD_5_	-0.124	−0.441	−0.136	0.494	1	-	-	-	-	-	-	-	-
	NH_4_^+^–N	−0.272	−0.689 *	0.187	0.790 **	0.731 *	1	-	-	-	-	-	-	-
	TP	−0.608	−0.748 *	−0.178	0.486	0.419	0.570	1	-	-	-	-	-	-
	Hg	0.258	−0.231	0.149	0.366	0.306	0.534	−0.183	1	-	-	-	-	-
	Pb	0.003	0.420	−0.613	−0.544	0.434	−0.104	−0.096	−0.045	1	-	-	-	-
	Volatile Phenols	−0.152	−0.670 *	−0.092	0.644 *	0.588	0.808 **	0.404	0.732 *	−0.010	1	-	-	-
	Petrol	−0.385	−0.117	−0.534	−0.091	0.689 *	0.286	0.549	−0.252	0.747 *	0.146	1	-	-
	*E. coli*	0.081	−0.474	0.734 *	0.786 **	0.094	0.598	0.219	0.291	−0.752 *	0.347	−0.429	1	-
	EC	−0.037	−0.786 **	0.435	0.721 *	0.165	0.558	0.510	0.413	−0.586	0.468	−0.184	0.562	1
Group C	Temperature	1	-	-	-	-	-	-	-	-	-	-	-	-
	DO	0.092	1	-	-	-	-	-	-	-	-	-	-	-
	pH	0.351	0.709 **	1	-	-	-	-	-	-	-	-	-	-
	COD_Mn_	0.495 *	−0.039	0.229	1	-	-	-	-	-	-	-	-	-
	BOD_5_	0.648 **	0.060	0.409	0.90 2 **	1	-	-	-	-	-	-	-	-
	NH_4_^+^–N	0.252	−0.338	−0.227	0.482 *	0.588 **	1	-	-	-	-	-	-	-
	TP	0.407	−0.136	0.384	0.607 **	0.665 **	0.299	1	-	-	-	-	-	-
	Hg	0.429 *	0.162	0.500*	0.195	0.320	−0.086	0.673 **	1	-	-	-	-	-
	Pb	0.078	−0.059	0.264	−0.438 *	−0.297	−0.318	0.218	0.468 *	1	-	-	-	-
	Volatile Phenols	0.567 **	−0.080	0.082	0.620 **	0.624 **	0.492 *	0.442 *	0.300	−0.232	1	-	-	-
	Petrol	0.504 *	−0.023	0.077	0.744 **	0.722 **	0.639 **	0.306	0.000	−0.510 *	0.866 **	1	-	-
	*E. coli*	0.271	−0.251	−0.101	0.566 **	0.549 **	0.664 **	0.295	−0.080	−0.419	0.774 **	0.805 **	1	-
	EC	−0.149	−0.349	−0.512*	0.335	0.009	0.139	−0.131	−0.421 *	−0.522 *	−0.032	0.109	0.205	1

* Correlation is significant at the 0.01 level and ** correlation is significant at the 0.05 level (2-tailed). DO = dissolved oxygen, COD_Mn_ = chemical oxygen demand, BOD_5_ = 5-day biochemical oxygen demand, TP = total phosphorus, EC = electrical conductivity.

**Table 3:** Loading of 13 water quality variables on significant varifactors (VFs) for group A, group B and group C.

Parameters	Group A					Group B						Group C				
	VF1	VF2	VF3	VF4	VF5	VF1	VF2	VF3	VF4	VF5	VF6	VF1	VF2	VF3	VF4	VF5
Temperature	−0.163	*0.625*	−0.076	*0.549*	−0.072	−0.042	−0.102	**−0.867**	−0.006	0.094	0.139	−0.119	−0.085	**0.836**	0.003	−0.033
DO	−0.290	**−0.741**	−0.117	0.058	−0.017	*−0.665*	−0.070	0.439	0.008	−0.030	0.404	−0.036	**0.870**	−0.142	−0.105	−0.017
pH	−0.122	0.021	0.035	**0.795**	−0.004	0.124	−0.033	−0.221	0.075	0.015	**0.851**	0.228	*0.739*	0.396	0.170	−0.058
COD_Mn_	**0.860**	0.041	0.032	0.021	0.097	*0.710*	0.285	0.178	−0.202	−0.047	0.184	**0.823**	−0.147	−0.003	−0.023	0.040
BOD_5_	**0.774**	0.061	−0.006	−0.02	0.053	0.226	**0.759**	0.187	0.153	0.074	0.207	*0.713*	0.312	0.181	0.158	0.078
NH_4_^+^–N	**0.785**	−0.108	0.125	−0.009	0.031	*0.684*	0.277	0.223	0.149	0.030	0.125	*0.590*	0.032	−0.439	0.243	−0.041
TP	0.370	0.108	*0.725*	−0.131	0.036	*0.564*	−0.178	0.045	*0.549*	−0.195	0.055	0.230	−0.150	−0.207	**0.754**	−0.158
Hg	0.080	0.387	−0.097	0.012	*0.631*	0.183	−0.050	0.093	−0.137	*0.718*	0.205	0.169	0.321	*0.557*	−0.107	−0.160
Pb	−0.067	−0.208	0.061	−0.035	**0.806**	−0.202	0.088	−0.120	0.189	**0.754**	−0.200	−0.086	0.189	0.310	0.176	−0.567
Volatile Phenol	0.455	−0.371	0.002	0.413	0.015	0.331	0.022	0.434	−0.122	0.245	−0.151	*0.510*	0.145	−0.242	−0.134	−0.142
Petrol	0.283	0.059	0.010	−0.042	−0.112	−0.035	**0.792**	−0.046	−0.183	−0.030	−0.247	*0.712*	0.023	0.078	−0.113	0.165
*E. coli*	−0.139	−0.026	**0.865**	0.116	−0.038	−0.128	0.013	−0.057	**0.882**	0.071	0.057	−0.295	0.137	0.112	**0.759**	0.180
EC	0.687	0.206	0.015	−0.241	−0.023	**0.793**	−0.105	0.016	−0.073	0.049	0.062	0.050	0.055	0.042	0.113	**0.794**
Eigenvalue	3.125	1.402	1.276	1.119	1.036	2.832	1.638	1.251	1.213	1.076	1.044	2.664	2.058	1.368	1.121	1.052
%Total Variance	24.036	10.786	9.817	8.608	7.971	21.781	12.599	9.620	9.331	8.280	8.033	20.494	15.835	10.523	8.623	8.095
Cumulative% Variance	24.036	34.822	44.639	53.246	61.217	21.781	34.381	44.001	53.331	61.611	69.645	20.494	36.329	46.852	55.474	63.570

*Note:* bold values indicate strong loadings and italic values indicate moderate loadings.

**Table 4:** Results for KMO and Bartlett’s sphericity test.

Periods		KMO	Bartlett’s Sphericity	Significance
Group A	HF Period	0.704	1487.28	0.000
	LF Period	0.702	771.018	0.000
	NF Period	0.582	545.283	0.000
Group B	HF Period	0.632	443.34	0.000
	LF Period	0.533	173.94	0.000
	NF Period	0.597	169.06	0.000
Group C	HF period	0.662	925.21	0.000
	LF period	0.571	556.05	0.000
	NF Period	0.484	251.88	0.000

**Table 5:** Source apportionment results for each period for the three different regions of pollution.

Periods		VF1	VF2	VF3	VF4	VF5
Group A	HF	Oxygen Consuming + Toxic Organic Pollution	Nutrient + Fecal Pollution	Heavy Metal Pollution (Hg)	Physicochemical Pollution	Heavy Metal Pollution (Pb)
	LF	Oxygen Consuming Organic Pollution	Physicochemical Pollution	Nutrient + Fecal Pollution	Toxic Organic Pollution	Heavy Metal Pollution (Pb)
	NF	Oxygen Consuming Organic Pollution	Nutrient + Fecal Pollution	Nature Pollution	Toxic Organic Pollution	Physicochemical Pollution
Group B	HF	Oxygen Consuming Organic Pollution	Oil Pollution	Nutrient + Fecal Pollution	Toxic Organic Pollution	Heavy Metal Pollution
	LF	Oxygen Consuming Organic + Oil Pollution	Fecal Pollution	Physicochemical Pollution	Heavy Metal Pollution (Pb)	-
	NF	Oxygen Consuming Organic Pollution + Fecal Pollution	Nutrient + Heavy Metal Pollution (Hg)	Physicochemical Pollution	Toxic Organic Pollution	-
Group C	HF	Oxygen Consuming Organic + Oil Pollution	Physicochemical Pollution	Nutrient + Fecal Pollution	Heavy Metal Pollution	-
	LF	Oil + Oxygen Consuming Organic Pollution	Heavy Metal Pollution (Hg)	Nutrient Pollution	Fecal Pollution	Heavy Metal Pollution (Pb)
	NF	Oxygen Consuming Organic + Toxic Organic + Oil Pollution	Heavy Metal Pollution (Hg)	Heavy Metal Pollution (Pb)	Nutrient + Fecal Pollution	Physicochemical Pollution

**Table 6:** Results from two different multivariate statistical models.

Groups	Principal Component Analysis (PCA)		Positive Matrix Factorization (PMF)	
	Source	Explained Variance (%)	Sources	Contribution to the Total Mass (%)
Group A	Cropland and Woodland Runoff	24.0	Cropland and Woodland Runoff	22.2
	Seasonal Changes	10.8	Domestic Wastewater	5.0
	Local Livestock Farms and Domestic Wastewater	9.8	Oil Pollution	4.3
	Physicochemical Source of the Variability	8.6	Seasonal Changes	19.0
	Mining Activity	8.6	Local Livestock Farms Wastewater, Mining Activity	49.5
	Others	38.1	-	-
Group B	Domestic Wastewater and Sewage Treatment Works	21.8	Industrial Sewage and Seasonal Change	33.2
	Oil Production and Petroleum Chemical Industry	12.6	Physicochemical Source	13.7
	Seasonal Changes	9.6	Local Livestock Farms and Domestic Wastewater	20.3
	Local Livestock Farms Wastewater	9.3	Gas-Fired and Cooking Water From Industry	28.0
	Industrial Sewage	8.3	Oil Production and Petroleum Chemical Industry	4.8
	Physicochemical Source	8.0	-	-
	Others	30.4	-	-
Group C	Oil production and Petroleum Chemical Industry	20.5	Domestic Wastewater	4.6
	Physicochemical Sources	15.8	Industrial Sewage and Seasonal Change	44.0
	Industrial Sewage	10.5	Industrial Sewage and Physicochemical Sources	39.0
	Local Livestock Farms and Domestic Wastewater	8.6	Local Livestock Farms Wastewater	2.1
	Seasonal Changes	8.1	Oil Production and Petroleum Chemical Industry	10.0
	Others	36.4	-	-

## Appendix A

**Table A1:** Analytical methods for water quality parameters of the Liao River system in Liaoning province, China.

Parameters	Analytical Methods	Limits of Detection
Temperature (°C)	Thermometer	-
DO (Mg/L)	Iodimetry	0.2
pH	Glass Electrode	-
COD_Mn_ (mg/L)	Potassium Permanganate Method	0.5
BOD_5_ (mg/L)	Dilution and Inoculation Test	0.5
NH_4_^+^–N (mg/L)	N−Reagent Colorimetry	0.05
TP (mg/L)	Ammonium Molybdate Spectrophotometry	0.01
Hg (mg/L)	Cold vapor Atomic Absorption Spectrometry	0.00005
Pb (mg/L)	Atomic Absorption Spectrophotometry	0.01
Volatile phenols (mg/L)	Spectrophotometric Determination with 4−Amino−Antipyrin	0.002
Petrol (mg/L)	Infrared Spectrophotometry	0.01
*E. coli* (num/L)	Manifold Zymotechnics Method/Filter Membrane Method	-
EC (ms/s)	Electrometric	-

**Table A2:** Statistical description of discharge (2000 and 2002–2010, Liaozhong Gauging Station) that coincides with each type of flow period.

Periods	Mean ± SD/(10^4^ m^3^)	Minimum/(10^4^ m^3^)	Maximum/(10^4^ m^3^)
HF	26,916 ± 41,834	692	247,276
LF	4058 ± 3396	79	13,867
NF	4521 ± 6129	128	28,927

*Note:* SD is the abbreviation of standard deviation.

**Table A3:** Comparison of mean values of water quality parameters by ANOVA between pollution regions and periods.

Parameters	Period Mean Value			Region Mean Value		
	HF	LF	NF	Group A	Group B	Group C
Temperature (°C)	20.0 ± 4.8c	5.4 ± 3.3a	7.6 ± 4.6b	14.5 ± 7.6a	16.1 ± 8.1b	17.2 ± 7.8b
DO (Mg/L)	6.78 ± 2.56a	8.06 ± 3.37b	8.00 ± 3.90b	8.30 ± 2.87c	4.37 ± 2.27a	7.00 ± 2.77b
pH	7.85 ± 0.44b	7.74 ± 0.42a	7.71 ± 0.41a	7.85 ± 0.39b	7.76 ± 0.50a	7.73 ± 0.46a
COD_Mn_ (mg/L)	22.49 ± 15.64a	30.07 ± 21.73b	22.98 ± 16.84a	19.24 ± 18.19a	36.13 ± 15.29c	27.58 ± 13.88b
BOD_5_ (mg/L)	5.57 ± 4.90a	8.61 ± 24.81b	5.92 ± 6.95a	5.22 ± 16.90a	8.87 ± 5.65c	7.07 ± 4.00b
NH_4_^+^–N (mg/L)	2.560 ± 3.897a	5.289 ± 6.443c	3.343 ± 4.083b	2.255 ± 4.182a	8.967 ± 5.800c	2.363 ± 2.762b
TP (mg/L)	0.266 ± 0.312a	0.417 ± 0.455b	0.244 ± 0.264a	0.214 ± 0.257a	0.693 ± 0.494c	0.232 ± 0.238b
Hg (mg/L)	0.000029 ± 0.000029a	0.000031 ± 0.000046a	0.000027 ± 0.000018a	0.000025 ± 0.000018a	0.000047 ± 0.000070c	0.000029 ± 0.000015b
Pb (mg/L)	0.00593 ± 0.00374a	0.00559 ± 0.00305a	0.00535 ± 0.00226a	0.00504 ± 0.00112a	0.00666 ± 0.00494b	0.00658 ± 0.00463b
Volatile phenols (mg/L)	0.0056 ± 0.0267a	0.0123 ± 0.0481c	0.0077 ± 0.0236b	0.0092 ± 0.0434c	0.0074 ± 0.0113b	0.0045 ± 0.0076a
Petrol (mg/L)	0.137 ± 0.220a	0.171 ± 0.120b	0.169 ± 0.291b	0.088 ± 0.206c	0.160 ± 0.221a	0.258 ± 0.233b
*E. coli* (num/L)	860,668 ± 5,082,132a	660,360 ± 3,203,293a	588,074 ± 3,250,654a	902,564 ± 5,118,360b	2280673 ± 6,333,392c	7131 ± 22,844a
EC (ms/s)	90.6 ± 225b	103.7 ± 122.0a	85.9 ± 76.6a	62.1 ± 34.1a	92.6 ± 33.3b	149.2 ± 332c

## References

[1] Varol M., Gökot B., Bekleyen A., Şen B. (2012). Spatial and temporal variations in surface water quality of the dam reservoirs in the Tigris River basin. Catena.

[2] Zhang Y., Guo F., Meng W., Wang X.Q. (2009). Water quality assessment and source identification of Daliao river basin using multivariate statistical methods. Environ. Monit. Assess..

[3] Huang F., Wang X., Lou L., Zhou Z., Wu J. (2010). Spatial variation and source apportionment of water pollution in Qiantang River (China) using statistical techniques. Water Res..

[4] Vega M., Pardo R., Barrado E., Debán L. (1998). Assessment of seasonal and polluting effects on the quality of river water by exploratory data analysis. Water Res..

[5] Chen J., Lu J. (2014). Effects of Land use, topography and socio-economic factors on river water Quality in a mountainous watershed with intensive agricultural production in East China. PLoS ONE.

[6] Zhao J., Fu G., Lei K., Li Y. (2011). Multivariate analysis of surface water quality in the three Gorges area of China and implications for water management. J. Environ. Sci. (China).

[7] Patel V., Parikh P. (2013). Assessment of seasonal variation in water quality of River Mini, at Sindhrot, Vadodara. Int. J. Environ. Sci..

[8] Chen X., Zhu L., Pan X., Fang S., Zhang Y., Yang L. (2015). Isomeric specific partitioning behaviors of perfluoroalkyl substances in water dissolved phase, suspended particulate matters and sediments in Liao River basin and Taihu Lake, China. Water Res..

[9] Li Y.L., Liu K., Li L., Xu Z.X. (2012). Relationship of land use/cover on water quality in the Liao River Basin, China. Proc. Environ. Sci..

[10] Statistical Bureau of Liaoning Province (SBLP) (2012). Liaoning Statistical Yearbook.

[11] Zhou L.J., Ying G.G., Zhao J.L., Yang J.F., Wang L., Yang B., Liu S. (2011). Trends in the occurrence of human and veterinary antibiotics in the sediments of the Yellow River, Hai River and Liao River in Northern China. Environ. Pollut..

[12] Lu J., Xu J., Guo C., Zhang Y., Bai Y., Meng W. (2014). Spatial and temporal distribution of polycyclic aromatic hydrocarbons (PAHs) in surface water from Liaohe River Basin, northeast China. Environ. Sci. Pollut. Res..

[13] Wang X.Q., Zhang Y.H. (2007). Pollution status and countermeasures of Liaohe Drainage Basin in Liaoning Province. Environ. Protect. Sci..

[14] Singh K.P., Malik A., Singh V.K., Mohan D., Sinha S. (2005). Chemometric analysis of groundwater quality data of alluvial aquifer of gangetic plain, North India. Anal. Chim. Acta.

[15] Alberto W.D., Del Pilar D.M., Valeria A.M., Fabiana P.S., Cecilia H.A., De Los Angeles B.M. (2001). Pattern recognition techniques for the evaluation of spatial and temporal variations in water quality. A case study: Suquía River Basin (Cordoba-Argentina). Water Res..

[16] Zhou F., Huang G.H., Guo H., Zhang W., Hao Z. (2007). Spatio-temporal patterns and source apportionment of coastal water pollution in eastern Hong Kong. Water Res..

[17] Wang J., Liu R., Wang H., Yu W., Xu F., Shen Z. (2015). Identification and apportionment of hazardous elements in the sediments in the Yangtze River Estuary. Environ. Sci. Pollut. Res..

[18] Selvaraju N., Pushpavanam S., Anu N. (2013). A holistic approach combining factor analysis, positive matrix factorization, and chemical mass balance applied to receptor modeling. Environ. Monit. Assess..

[19] Lu P., Mei K., Zhang Y., Liao L., Long B., Dahlgren R.A., Zhang M. (2011). Spatial and temporal variations of nitrogen pollution in Wen-Rui Tang River watershed, Zhejiang, China. Environ. Monit. Assess..

[20] Zhou F., Guo H., Liu Y., Jiang Y. (2007). Chemometrics data analysis of marine water quality and source identification in Southern Hong Kong. Mar. Pollut. Bull..

[21] Gao X., Zhang Y., Ding S., Zhao R., Meng W. (2015). Response of fish communities to environmental changes in an agriculturally dominated watershed (Liao River Basin) in Northeastern China. Ecol. Eng..

[22] Xu X.L., Pang Z.G., Yu X.F. (2005). Spatial-Temporal Pattern Analysis of Land Use/Cover Change: Methods &Applications.

[23] Liu J.Y. (1996). Macro-Scale Survey and Dynamic Study of Natural Resources and Environment of China by Remote Sensing.

[24] Zhu D. (2007). Dictionary of the Chinese River.

[25] Yue F., Li S., Liu C., Zhao Z., Hu J. (2013). Using dual isotopes to evaluate sources and transformation of nitrogen in the Liao River, Northeast China. Appl. Geochem..

[26] Bai Y., Meng W., Xu J., Zhang Y., Guo C. (2014). Occurrence, distribution and bioaccumulation of antibiotics in the Liao River Basin in China. Environ. Sci. Proc. Impacts.

[27] State Environmental Protection Administration (SEPA) (2002). Water and Wastewater Analysis Method.

[28] Data Center for Resources and Environmental Sciences, Chinese Academy of Sciences (RESDC). http://www.resdc.cn.

[29] Qu W., Kelderman P. (2001). Heavy metal contents in the Delft canal sediments and suspended solids of the river Rhine: Multivariate analysis for source tracing. Chemosphere.

[30] Lattin J.M., Carroll J.D., Green P.E. (2003). Analyzing Multivariate Data.

[31] Medina-Gómez I., Herrera-Silveira J.A. (2003). Spatial characterization of water quality in a karstic coastal lagoon without anthropogenic disturbance: a multivariate approach. Estuar. Coast. Shelf Sci..

[32] McKenna J. (2003). An enhanced cluster analysis program with bootstrap significance testing for ecological community analysis. Environ. Model. Softw..

[33] Shrestha S., Kazama F. (2007). Assessment of surface water quality using multivariate statistical techniques: A case study of the Fuji River Basin, Japan. Environ. Model. Softw..

[34] Simeonova P., Simeonov V., Andreev G. (2003). Water quality study of the Struma river basin, Bulgaria (1989–1998). Open Chem..

[35] Johnson R.A., Wichern D.W. (2002). Applied Multivariate Statistical Analysis.

[36] Zhou F., Liu Y., Guo H. (2007). Application of multivariate statistical methods to water quality assessment of the watercourses in Northwestern New Territories, Hong Kong. Environ. Monit. Assess..

[37] Chen J., Lu J. (2014). Establishment of reference conditions for nutrients in an intensive agricultural watershed, Eastern China. Environ. Sci. Pollut. Res..

[38] Pekey H., Karakaş D., Bakoğlu M. (2004). Source apportionment of trace metals in surface waters of a polluted stream using multivariate statistical analyses. Mar. Pollut. Bull..

[39] Brūmelis G., Lapiņa L., Nikodemus O., Tabors G. (2000). Use of an artificial model of monitoring data to aid interpretation of principal component analysis. Environ. Model. Softw..

[40] Helena B., Pardo R., Vega M., Barrado E., Fernandez J.M. (2000). Temporal evolution of groundwater composition in an alluvial aquifer (Pisuerga River, Spain) by principal component analysis. Water Res..

[41] Hannaford J., Buys G. (2012). Trends in seasonal river flow regimes in the UK. J. Hydrol..

[42] Duan B., Liu F., Zhang W., Zheng H., Zhang Q., Li X., Bu Y. (2015). Evaluation and source apportionment of heavy metals (HMs) in sewage sludge of municipal wastewater treatment Plants (WWTPs) in Shanxi, China. Int. J. Environ. Res. Public Health.

[43] Yang L., Zhu L., Liu Z. (2011). Occurrence and partition of perfluorinated compounds in water and sediment from Liao River and Taihu Lake, China. Chemosphere.

[44] Wang L., Ying G.G., Zhao J.L., Liu S., Yang B., Zhou L.J., Tao R., Su H.C. (2011). Assessing estrogenic activity in surface water and sediment of the Liao River system in northeast China using combined chemical and biological tools. Environ. Pollut..

[45] Kowalkowski T., Zbytniewski R., Szpejna J., Buszewski B. (2006). Application of chemometrics in river water classification. Water Res..

[46] Wang L., Ying G.G., Zhao J.L., Yang X.B., Chen F., Tao R., Liu S., Zhou L.J. (2010). Occurrence and risk assessment of acidic pharmaceuticals in the Yellow River, Hai river and Liao River of North China. Sci. Total Environ..

[47] Men B., He M., Tan L., Lin C., Quan X. (2009). Distributions of polycyclic aromatic hydrocarbons in the Daliao River Estuary of Liaodong Bay, Bohai Sea (China). Mar. Pollut. Bull..

[48] Kannel P.R., Lee S., Lee Y.S. (2008). Assessment of spatial-temporal patterns of surface and ground water qualities and factors influencing management strategy of groundwater system in an urban river corridor of Nepal. J. Environ. Manag..

[49] Jiang J., Wang J., Liu S., Lin C., He M., Liu X. (2013). Background, baseline, normalization, and contamination of heavy metals in the Liao River watershed sediments of China. J. Asian Earth Sci..

[50] Ke X., Gao L., Huang H., Kumar S. (2015). Toxicity identification evaluation of sediments in Liaohe River. Mar. Pollut. Bull..

[51] Maillard P., Santos N.A. (2008). A spatial-statistical approach for modeling the effect of non-point source pollution on different water quality parameters in the Velhas river watershed—Brazil. J. Environ. Manag..

[52] Bhuiyan M.A.H., Dampare S.B., Islam M.A., Suzuki S. (2014). Source apportionment and pollution evaluation of heavy metals in water and sediments of Buriganga River, Bangladesh, using multivariate analysis and pollution evaluation indices. Environ. Monit. Assess..

[53] Khan M.F., Hirano K., Masunaga S. (2012). Assessment of the sources of suspended particulate matter aerosol using US EPA PMF 3.0. Environ. Monit. Assess..

[54] Huang L., Li Y., Zhang Y., Guan Y. (2014). A simple method to separate phosphorus sorption stages onto solid mediums. Ecol. Eng..

[55] Huang L., Zhang Y., Shi Y., Liu Y., Wang L., Yan N. (2015). Comparison of phosphorus fractions and phosphatase activities in coastal wetland soils along vegetation zones of Yancheng National Nature Reserve, China. Estuar. Coast. Shelf Sci..

[56] Bu H., Wan J., Zhang Y., Meng W. (2013). Spatial characteristics of surface water quality in the Haicheng river (Liao River Basin) in Northeast China. Environ. Earth Sci..

[57] Yao H., Qian X., Gao H., Wang Y., Xia B. (2014). Seasonal and spatial variations of heavy metals in two typical Chinese Rivers: Concentrations, environmental risks, and possible sources. Int. J. Environ. Res. Public Health.

